# Hypocomplementemic Urticarial Vasculitis Syndrome: A Rare Form of Vasculitis

**DOI:** 10.7759/cureus.78227

**Published:** 2025-01-30

**Authors:** Saurabh Kumar Singh, Mohit Gupta, Shilpi Rani, Pratap Singh

**Affiliations:** 1 Cardiology, Vardhman Mahavir Medical College and Safdarjung Hospital, New Delhi, IND; 2 Medicine, Atal Bihari Vajpayee Institute of Medical Sciences and Dr. Ram Manohar Lohia Hospital, New Delhi, IND; 3 Internal Medicine, Vardhman Mahavir Medical College and Safdarjung Hospital, New Delhi, IND

**Keywords:** dapsone, hypocomplementemic urticarial vasculitis syndrome, urticaria, vasculitis, young female patient

## Abstract

Hypocomplementemic urticarial vasculitis syndrome (HUVS) is a rare autoimmune disorder characterized by recurrent urticarial lesions and acquired hypocomplementemia with systemic manifestations. Systemic involvement can either be present at the onset of disease or develop later. Here, we present a rare case of a 22-year-old female, who initially presented with generalized rash and was eventually diagnosed with HUVS. She responded well to dapsone. This article emphasizes the importance of a comprehensive review of systemic manifestations accompanying urticaria.

## Introduction

Urticarial vasculitis (UV) represents a spectrum of diseases characterized by urticaria and histopathologic evidence of leukocytoclastic vasculitis. It is a type III hypersensitivity reaction mediated by immune complex deposition on capillaries and postcapillary venules. The antigens eliciting the formation of antibodies are not known. Medications and viruses (hepatitis B & C) have been implicated as the target antigens in some cases [1]. In some patients with UV, particularly those with hypocomplementemic urticarial vasculitis syndrome (HUVS), a collagen-like region on C1q has been demonstrated as an antigenic target [2].

UV represents a spectrum of diseases ranging from urticaria with minimal vasculitis to organ-threatening systemic vasculitis. Some patients have low complement levels, a feature that is associated with severe disease and systemic involvement, known as HUVS. In the absence of hypocomplementemia, the disease tends to be milder and is referred to as normocomplementemic urticarial vasculitis (NUV) [3]. UV is a rare disorder. A Swedish study ascertained an incidence of HUVS of 0.7 per million inhabitants being significantly higher among females than males [4]. HUVS was first described in 1973 by Mcduffie et al., thus, it is also known as Mcduffie syndrome [5]. Diagnostic criteria require the presence of both major criteria plus at least two minor criteria for definitive diagnosis [6].

Hypocomplementemic urticarial vasculitis (HUV) is the term used to describe patients with UV and hypocomplementemia not meeting the diagnostic criteria for HUVS. Multi-organ involvement is generally seen, which includes the skin (100%), joints (70%), kidney (50%), gastrointestinal system (30%), lungs (20%), and eyes (10%) [7]. Treatment is mainly immunosuppressive or immunomodulatory agents depending on disease severity and systemic manifestations.

## Case presentation

**Figure 1:**
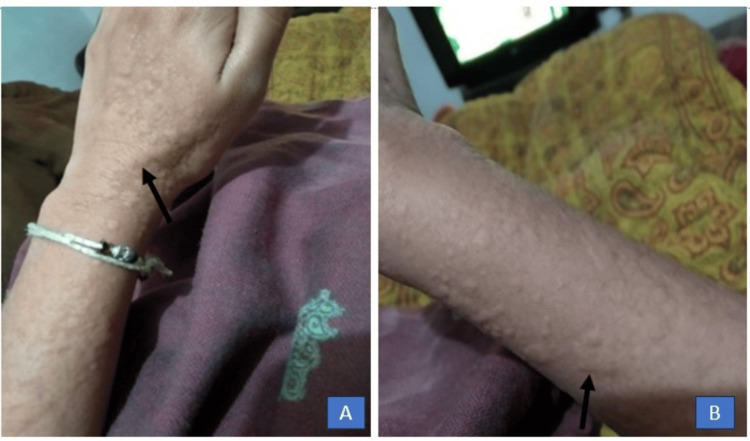
Wheals on the dorsal aspect of the right forearm and hand (A) and right leg (B).

A 22-year-old female with no previous comorbidities presented with complaints of painless rash on and off for six months, initially skin colored associated with itching and palpable all over the body, which remained for one to two days and then disappeared completely without any systemic features, as shown in Figure 1.

**Figure 2:**
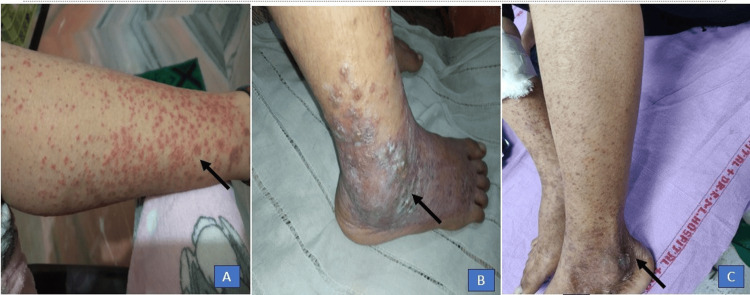
Fresh eruption of rashes showing petechiae and palpable purpura on the right lower limb (A). Palpable purpuric lesions along with intact vesicles and bullae over the background of diffuse erythema, predominantly lower one-third of the leg and dorsum of feet (B). Disappearance of rashes with residual hyperpigmentation (C).

Roughly four months after the initial skin manifestations, the patient developed palpable purpura and petechiae that were non-blanching and painful (Figure 2A). Some of the lesions coalesced to form erythematous plaque and bullae predominantly over the dependent areas of bilateral lower limbs, more over the dorsum of feet and lower one-third of the leg (Figure 2B). Over time, the rash progressed to bilateral lower limbs, trunk, buttocks, and upper limbs. The rash resolved over five to six days with residual hyperpigmentation (Figure 2C). These episodes gradually increased in frequency.

It was accompanied by joint pain and swelling, involving both large and small joints (knee, ankle, elbow, hand, and feet), migratory with morning stiffness of more than 30 minutes, and resolved with the disappearance of the rash. The patient also noticed blood in urine and stool and severe spasmodic pain in the abdomen; three episodes in the last one month associated with headache and malaise for two months, for which she took over-the-counter medication during acute episodes. There was no history of fever, cough, shortness of breath, decrease in urine output, or chronic drug intake prior to illness. No history of oral ulcers, photosensitivity, Raynaud’s phenomenon, or alopecia was noted.

The examination was remarkable for rashes and tenderness present in joints (knee, ankle, and wrist). Abdominal examination suggested diffuse tenderness and no hepatosplenomegaly was noted. Blood investigations were done, as shown in Table 1.

**Table 1:** Laboratory findings. TLC: total leukocyte count; ESR: erythrocyte sedimentation rate; CRP: C-reactive protein; AST: aspartate aminotransferase; ALT: alanine aminotransferase; ALP: alkaline phosphatase; ANA: antinuclear antibody; ENA: extractable nuclear antigens; c-ANCA: cytoplasmic anti-neutrophil cytoplasmic antibodies; p-ANCA: perinuclear anti-neutrophil cytoplasmic antibodies; IgA: immunoglobulin A; IgG: immunoglobulin G; IgM: immunoglobulin M; IgE: immunoglobulin E; HBsAg: hepatitis B surface antigen; anti-HCV: anti-hepatitis C antibody; anti-HIV: anti-human immunodeficiency virus antibody; RBC: red blood cells.

Routine blood investigations	Patient’s value	Normal range
Hemoglobin (g/dL)	11.4	11.5-17
TLC (cells/mm^3^)	8700	4-11
Platelets (L/mm3)	2.9	1.5-4.5
ESR (mm/hour)	35	<15
CRP	Positive	
Urea (mg/dl)	13	17-43
Creatinine	0.4	0.6-1.3
Total protein (gm/dl)	7	6-8.5
Albumin (gm/dl)	4.5	3.5-5.5
AST (U/L)	25	10-35
ALT (U/L)	36	10-45
ALP (U/L)	66	40-128
Total bilirubin (mg/dl)	0.6	0.3-1.2
Sodium (mmol/L)	140	136-145
Potassium (mmol/L)	4.1	3.5-5.5
Immunological results		
ANA	Negative	
ENA	Negative	
p-ANCA	Negative	
c-ANCA	Negative	
C4 (mg/dl)	8	15-40
C3 (mg/dl)	43	65-190
Cryoglobulins	Negative	
IgA (mg/dl)	268	70-400
IgM (mg/dl)	167	40-230
IgG (mg/dl)	946	700-1600
IgE (mg/dl)	476	150-300
Viral serology		
HBsAg/anti-HCV/anti-HIV	Non-reactive	
Urine analysis		
RBC	15-20/hpf	
WBC	1-2/hpf	
Protein	Negative	
Glucose	Negative	

**Figure 3:**
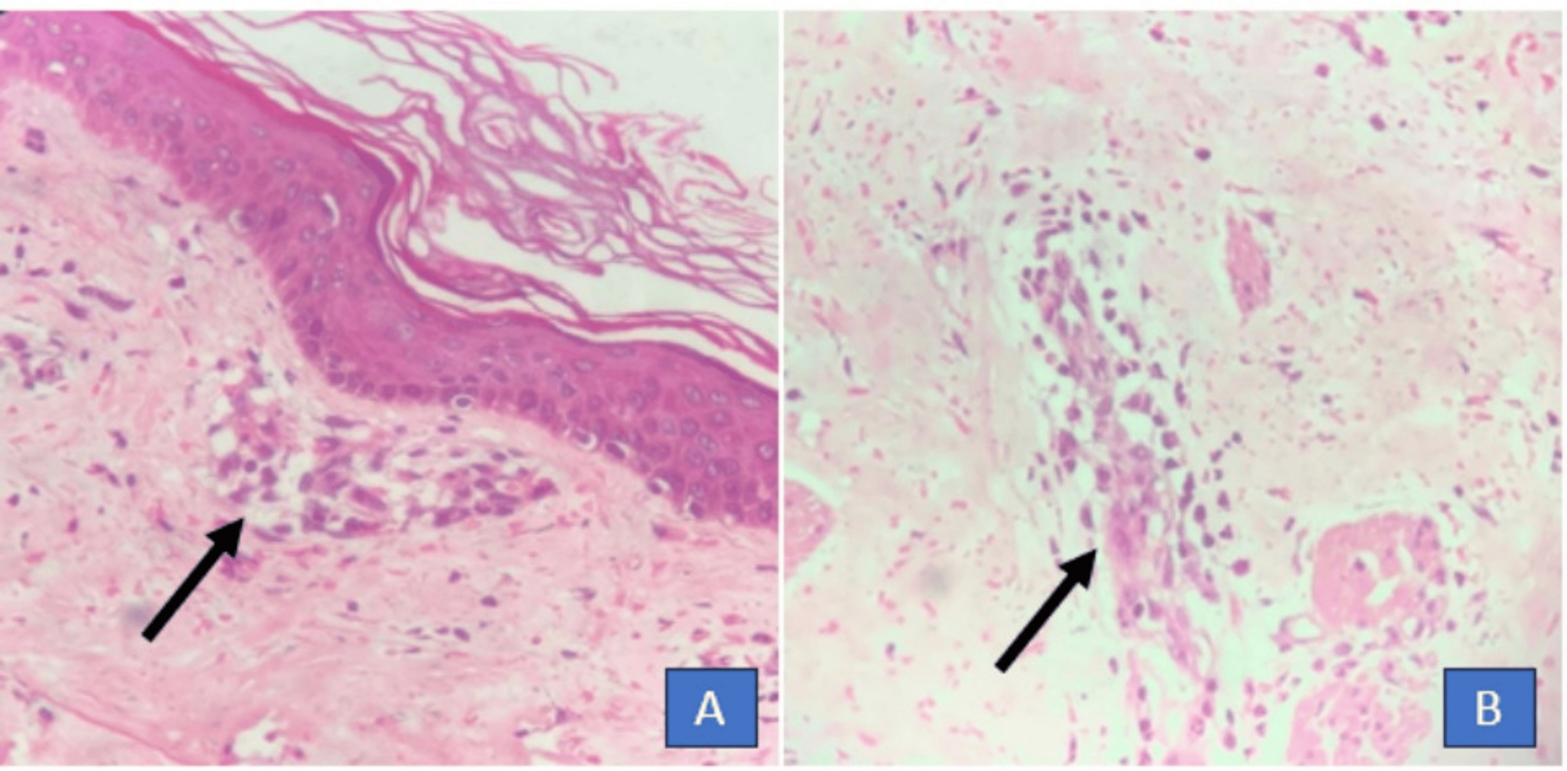
Perivascular inflammatory infiltrate comprising predominantly neutrophils with red cell extravasation and perivascular edema suggestive of leukocytoclastic vasculitis using hematoxylin and eosin stain (H&E) (A) and high-power view (100x magnification) showing numerous neutrophils and lymphocytes in the dermis (B). Methyl violet stain was positive in mast cells, which confirms urticarial vasculitis.

A skin biopsy was done, which was suggestive of leukocytoclastic vasculitis, and methyl violet stain was positive in mast cells, which confirms urticarial vasculitis, as shown in Figure 3.

**Figure 4:**
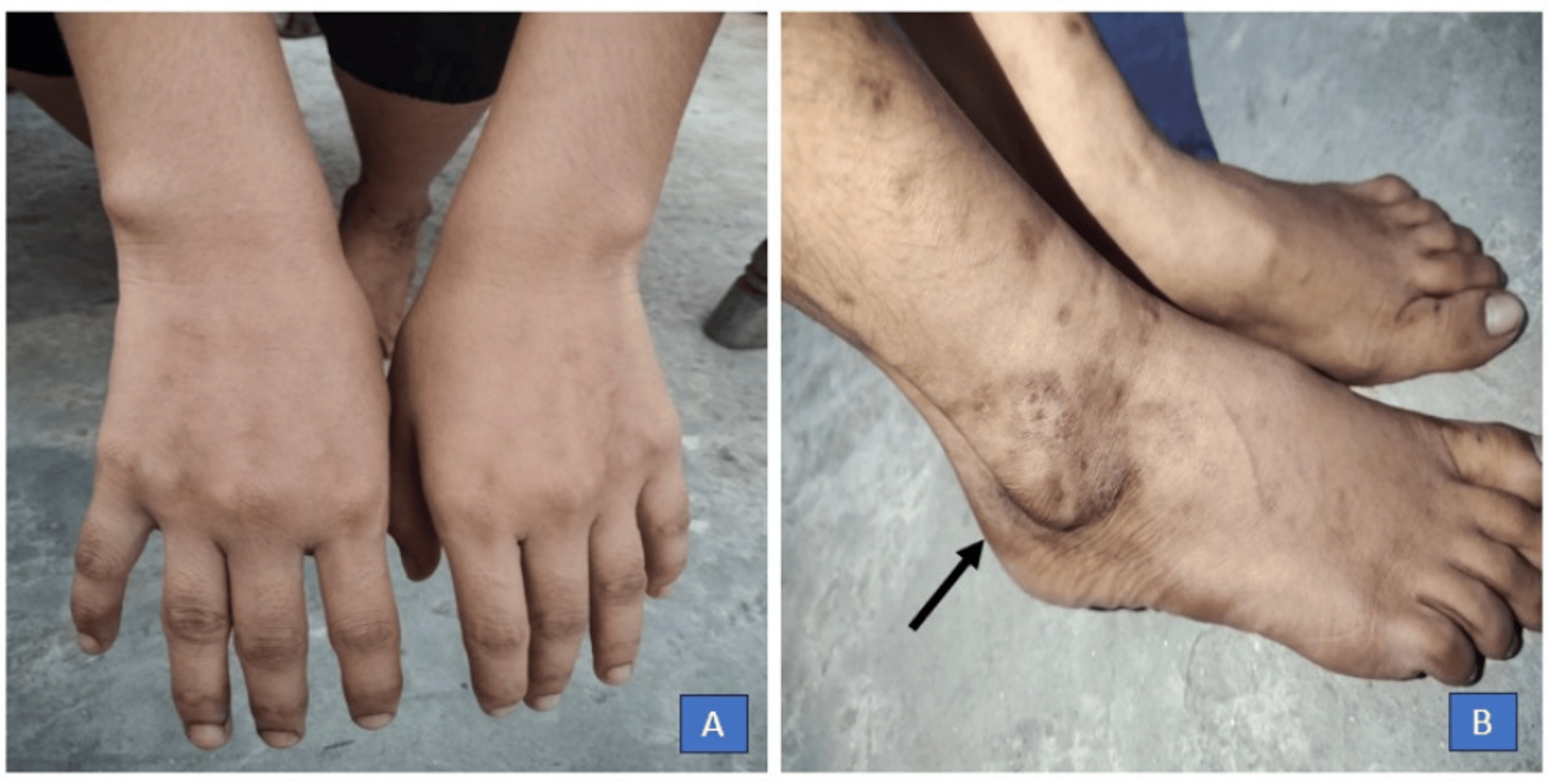
Post treatment with dapsone. (A) Complete clearing of lesions and (B) residual hyperpigmentation.

Finally, a diagnosis of hypocomplementemic urticarial vasculitis syndrome was made. She was given dapsone 100 mg once a day and responded well, as shown in Figure 4.

## Discussion

UV is a rare form of leukocytoclastic vasculitis. HUV was included in the Chapel Hill Consensus Conference (2012) in the category of small vessel vasculitis. Pathogenesis of HUVS is mainly due to the deposition of immune complexes in the vessel wall. Although the exact mechanism of this autoimmune phenomenon is unclear, in a few cases, C1q has been implicated as an antigenic target [2]. Genetic factors also play a major role in the pathogenesis. Familial forms of HUVS and HUVS associated with systemic lupus erythematosus (SLE) have been associated with mutations in DNASE1L3 [8].

It occurs mainly in young females, with a female-to-male ratio of 8:1 [7]. Multisystem involvement is generally seen, with the urticarial wheal being a prominent dermatological finding. Arthralgia or arthritis is a common finding accompanying the skin manifestations. Joint pain is usually transient and migratory, involving both large and small joints (as in our case). Renal disease is characterized by proteinuria and hematuria. Proliferative glomerulonephritis, crescentic glomerulonephritis, membranoproliferative, and tubulointerstitial nephritis have also been reported in HUVS [9]. Our patient had a history of gross hematuria without proteinuria or AKI. Gastrointestinal symptoms, mainly due to GI vasculitis, are seen in one-third of patients and include recurrent abdominal pain, nausea, vomiting, and diarrhea (as in our case) [10].

Pulmonary involvement can cause cough, dyspnea, hemoptysis, chronic obstructive pulmonary disease (COPD), and asthma, as seen in 20% of patients with HUVS and 5% with normocomplementemic UV [11]. Common ophthalmological findings include episcleritis, uveitis, and conjunctivitis. CNS involvement includes pseudotumor cerebri, aseptic meningitis, cranial nerve palsies, peripheral neuropathy, and transverse myelitis. Cardiac involvement is uncommon [12].

Laboratory investigations mainly include elevated erythrocyte sedimentation rate, hypocomplementemia (low C1q, C3, and C4), and anti-C1q antibodies in serum. Exclusion criteria were fulfilled in our case, which included cryoglobulinemia, elevated titer of anti-ds DNA antibodies, anti-Smith antibodies, hepatitis B virus antigenemia, and high titer of antinuclear antibodies (ANA). ANA can be positive in 50% of cases, but in our case, it was negative [7]. The histopathological evaluation of a skin lesion is considered the gold standard for diagnosing cutaneous vasculitis, including UV, which was consistent with our biopsy finding. A final diagnosis of HUVS was made (as both major and three minor criteria were present) as per Schwartz's criteria, as shown in Table 2 [6].

**Table 2:** Schwartz criteria for the diagnosis of hypocomplementemic urticarial vasculitis syndrome (HUVS). Source: Schwartz et al. [6].

Major criteria (must have both)	Minor criteria (must have two)
Urticarial skin lesions	Dermal venulitis
Low level of serum complements	Arthritis
	Glomerulonephritis
	Episcleritis or uveitis
	Recurrent abdominal pain
	C1q precipitin in plasma

Treatment of UV is based on the severity of the disease. For mild diseases (urticaria and arthralgia), antihistamines and nonsteroidal anti-inflammatory drugs (NSAIDs) are given. Dapsone with or without glucocorticoids are initial therapy for mild to moderate disease (no life-threatening disease like renal failure). Dapsone 50-300 mg/day is quite safe with few adverse effects like hemolysis and methemoglobinemia, rarely it can cause leukopenia, cholestatic jaundice, and peripheral neuropathy [8]. If dapsone is contraindicated, colchicine (1-1.2 mg/day) or hydroxychloroquine (HCQ) (200-400 mg/day) are an alternative. Colchicine commonly causes GI side effects like diarrhea, nausea, vomiting, and abdominal pain, and rarely causes neuromuscular toxicity and myelosuppression. HCQ is contraindicated with QT-prolonging drugs and the adverse effects are retinal damage, agranulocytosis, aplastic anemia, ataxia, peripheral neuropathy, and cardiomyopathy. These adverse effects are dose-related and usually occur when the cumulative dose exceeds 5 mg/kg actual weight. The duration of therapy is not fixed and depends on clinical response and serum complement levels. For severe systemic disease with life-threatening manifestations, combination therapy - glucocorticoids plus other agent (mycophenolate mofetil, methotrexate, azathioprine, cyclosporine) - is used. Biologicals can be used in refractory cases [9-11]. Our patient had UV with systemic manifestations, without any life-threatening condition, so the patient was given dapsone and responded dramatically.

## Conclusions

UV is a complicated disease with an unpredictable outcome. HUVS is a rare disease with systemic involvement and diagnosis is made based on clinical, laboratory, and histological examinations. The prognosis mainly depends on the severity of systemic manifestations and is influenced primarily by the severity of lung, heart, and kidney disease. When pulmonary involvement is present, COPD is the major cause of death. Therefore, early diagnosis and prompt treatment are the key.
